# Surgical audience focused on multimodality treatment for lung cancer

**DOI:** 10.1093/icvts/ivaf151

**Published:** 2025-07-08

**Authors:** Francesca Rita Ogliari, Sarah Debakker, Tom van Zwieten, Koen J Hartemink, Jonas Willmann, Anna M Sadowska, Lizza Hendriks

**Affiliations:** Department of Medical Oncology, IRCCS Ospedale San Raffaele, Milan, Italy; Università Vita-Salute San Raffaele, Milan, Italy; Department of Pulmonary Diseases, GROW Research Institute for Oncology and Reproduction, Maastricht University Medical Center+, Maastricht, The Netherlands; Department of Surgery, Netherlands Cancer Institute—Antoni van Leeuwenhoek Hospital, Amsterdam, The Netherlands; Department of Surgery, Netherlands Cancer Institute—Antoni van Leeuwenhoek Hospital, Amsterdam, The Netherlands; Department of Thoracic Oncology, Leiden University Medical Center, Leiden, The Netherlands; Department of Radiation Oncology, University Hospital Zurich, University of Zurich, Zurich, Switzerland; Department of Medical Physics, Memorial Sloan Kettering Cancer Center, New York, NY, USA; Department of Pulmonary Diseases, GROW Research Institute for Oncology and Reproduction, Maastricht University Medical Center+, Maastricht, The Netherlands; Department of Pulmonary Diseases, GROW Research Institute for Oncology and Reproduction, Maastricht University Medical Center+, Maastricht, The Netherlands

## INTRODUCTION

The landscape of early-stage non-small-cell lung cancer (NSCLC) has been rapidly evolving in the last years due to recently approved treatment strategies encompassing immunotherapy and targeted therapies in the peri-operative setting, both for oncogene and non-oncogene addicted tumours.

Historically, the only recommended post-operative systemic treatment was adjuvant platinum-based chemotherapy (PBC) for patients with tumours ≥4 cm and/or with positive lymph nodes in the surgical specimen. However, the magnitude of benefit for adjuvant chemotherapy is scarce, achieving an overall improvement in survival of 4–5% at 5 years. Additionally, despite being less common in clinical practice, neoadjuvant PBC resulted in similar survival benefits, although approximately 10% of patients did not proceed to surgery.

Given the relatively small advantage in terms of overall survival (OS) with standard PBC and the rapidly evolving field of systemic therapies with high response rate and long-term disease control in the metastatic scenario, the treatment paradigm for early-stage NSCLC has shifted towards more efficient peri-operative treatments aiming to improve the rate of cured patients.

**Figure 1:**
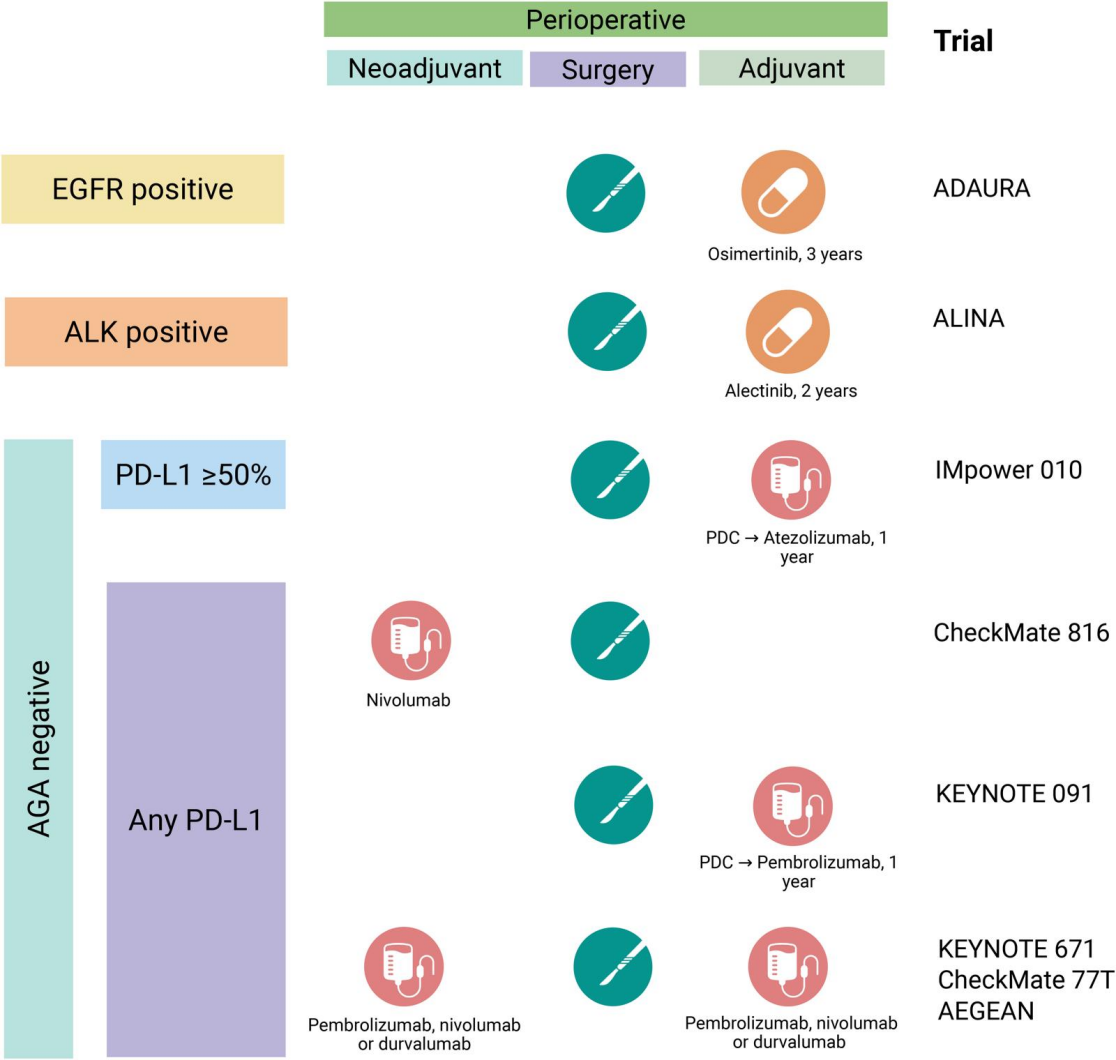
Graphical abstract of current treatment options for early-stage non-small-cell lung cancer as per Food and Drug Administration approvals in May 2025. To note, the Checkmate 77 T combination is not approved by the European Medicines Agency (EMA). For other selection criteria of neoadjuvant chemo-immunotherapy, see EMA labels. AGA: actionable genomic alterations; PDC: platinum-doublet chemotherapy

With this plethora of new treatment options, determining the optimal, personalized treatment approach for patients with resectable NSCLC becomes increasingly challenging. In this editorial, we explore the current standard of care (as summarized in Fig. 1) and its future challenges.

## PATIENT SELECTION

### Stage I

Surgery has always been the mainstay of treatment for patients with stage I and II NSCLC. For patients considered unfit for surgery or who prefer a non-surgical approach, stereotactic ablative body radiotherapy (SABR) is considered an alternative treatment in stage I NSCLC [1]. Long-term prospective data comparing surgery and SABR are lacking, with randomized controlled trials facing an early closure because of poor accrual [2], but available retrospective evidence tends to show a higher rate of regional recurrences for patients treated with SABR [3].

In two trials [4, 5], sublobar resection emerged as an acceptable surgical modality for small peripheral tumours (<2 cm) with comparable outcomes in terms of OS. However, local recurrence rates are higher with this approach, and lung function sparing is only limited when compared to lobectomy, leaving open questions about the role of sublobar resections in early-stage NSCLC.

### Stages II–III

For patients with resectable stage II and III NSCLC, treatment options have massively increased, with the approval of several immunotherapeutic agents plus PBC in the neoadjuvant or peri-operative setting, as well as the approval of adjuvant tyrosine kinase inhibitors (TKIs) for patients with epidermal growth factor receptor (*EGFR*) or anaplastic lymphoma kinase *(ALK*) positive NSCLC. Notably, in these trials, tumours had to be considered resectable upfront by multidisciplinary evaluation.

Staging should be done with computed tomography (CT) scan and fluorodeoxyglucose positron emission tomography, as well as brain imaging, preferably magnetic resonance imaging. Although mediastinal staging is recommended in clinical guidelines, staging requirements differed across trials and were often not mandatory.

Baseline molecular profiling was often mandatory in patients with non-squamous histology to exclude tumours that harbored an alteration in *EGFR* or *ALK* genes, while other more rare molecular alterations were not mentioned in the eligibility criteria. On the other hand, in the immunotherapy trials, there was no selection based on programmed-death ligand-1 (PD-L1) expression, but it was often used as a stratification factor for further efficacy analyses.

## IMMUNOTHERAPY-BASED STRATEGIES

### Neoadjuvant-only strategy

The role of immunotherapy in the neoadjuvant setting has been widely explored in the last decade. Firstly, a pilot study showed the safety and feasibility of two preoperative doses of nivolumab (programmed-death-1 or PD-1 inhibitor) in 21 patients with resectable stage I–III NSCLC [6]. The 5-year results of this trial showed an OS rate of 80% and a relapse-free survival rate of 60%. Of note, only one of nine patients with a major pathological response (MPR) and none of the two patients with a pathological complete response (pCR) had a disease recurrence at 5 years [7]. To further improve the outcomes of neoadjuvant immunotherapy, in the randomized phase III CheckMate-816 trial, patients with stage IB-IIIA NSCLC (TNM7) were randomized between three cycles of PBC-placebo (standard arm) or PBC-nivolumab (experimental arm). The primary end-points were event-free survival (EFS) and rate of pCR. At the final data cut-off, both primary end-points were met: EFS was 43.8 versus 18.4 months (hazard ratio [HR] 0.66, 95% confidence interval [CI] 0.49–0.90) and the rate of pCR was 24.0% versus 2.2% for experimental and standard arm, respectively. Interestingly, at 4-year follow-up, OS was not reached in both arms, but a positive trend was in favour of nivolumab (4-year OS rate 71% versus 58%), especially for the patients obtaining a pCR (HR 0.08, 95% CI 0.02–0.34) [8]. In was recently reported that the trial was also positive for OS, with a 5-year OS of 65% (nivolumab-chemotherapy) versus 55% (chemotherapy) [9].

### Adjuvant-only strategy

Three randomized phase III trials have evaluated the role of adjuvant immunotherapy in radically resected NSCLC. In the IMpower-010 trial, patients with resected stage IB-IIIA PD-L1 ≥ 1% NSCLC (TNM7), treated with adjuvant PBC, were randomized to 1-year atezolizumab (PD-L1 inhibitor) versus best-supportive care. Primary end-point was investigator-assessed DFS. A significant improvement in DFS was shown for the immunotherapy arm (57.4 versus 40.8 months, HR 0.83, 95% CI 0.69–1.00), as well as a preliminary trend for improved OS, especially driven by the subgroup with tumours with PD-L1 tumor proportion score (TPS) of 50% or higher (HR 0.48 for DFS, 95% CI 0.32–0.72; HR 0.47 for OS, 95% CI 0.28–0.77) [10, 11].

In the randomized phase III PEARLS/KEYNOTE-091 trial, patients with resected stage IB-IIIA NSCLC (TNM7) treated with or without adjuvant PBC were randomized to 1-year pembrolizumab (PD-1 inhibitor) or placebo. Dual primary end-points were DFS in the overall population (PD-L1 unselected) and in the population with PD-L1 TPS of ≥50%. In the overall population, median DFS was 53.6 versus 42.0 months for pembrolizumab versus placebo, respectively (HR 0.76, 95% CI 0.63–0.91) [12].

The randomized phase III BR31 trial evaluated 1 year of adjuvant durvalumab (PD-L1 inhibitor) versus placebo in this same setting of radically resected stage IB (≥4 cm), II or IIIA NSCLC (TNM7). Results presented at ESMO 2024 were negative for the primary end-point: in patients with a PD-L1 expression of 25% or higher on tumor cells, DFS was 70 months (durvalumab) and 60 months (placebo), respectively (HR 0.94, 95% CI 0.71–1.25) [13].

### Peri-operative strategies

Multiple randomized phase III trials evaluated the role of peri-operative immunotherapy-based regimens. Trial designs were slightly different, for example, according to stratification factors, platinum regimens allowed and total number of treatment cycles. In a meta-analysis from Nuccio *et al.*, immunotherapy combinations showed a significant improvement in EFS (HR 0.67, 95% CI 0.52–0.85) and pCR (HR 0.55, 95% CI 0.44–0.69) compared to chemotherapy-only strategies, and this benefit was consistent across all subgroups of patients irrespective of PD-L1 expression [14]. Across all trials, approximately 25% of enrolled patients were stage IIIB according to TNM 8th edition (i.e. T3N2 or T4N2). In the metanalysis, there was no difference in terms of EFS between stage IIIA or stage IIIB, suggesting that surgery could be offered to adequately selected patients with stage IIIB disease upon appropriate multidisciplinary evaluation [14].

Up to date, patient selection for the best treatment (neoadjuvant only, peri-operative or adjuvant only) is still a matter of debate. Systemic treatment upfront, such as in the neoadjuvant or peri-operative approach, is immediately effective against micro-metastases, priming the immune system and potentially fostering better resectability. On the other hand, immune-related adverse events or early disease progression can delay or abrogate surgery. Contrarily, the adjuvant-only strategy relies on extended pathological data coming from surgical samples and exploits the radicality of surgery without delays, but, in this setting, patients’ adherence to treatment tends to be lower.

Additionally, it is not known which patients need adjuvant immunotherapy after having received neoadjuvant chemo-immunotherapy. At a patient’s level, MPR and, above all, pCR are strong positive predictive factors, resulting in better EFS across all treatment arms [15]. These patients are therefore expected to achieve good outcomes irrespective of treatment strategy (neoadjuvant versus peri-operative), but cross-trial comparisons are weak and randomized data are lacking. Moreover, PD-L1 expression alone does not work well for prediction in this context. It does not correlate strongly with pCR (although the rate of pCR was doubled in PD-L1-positive tumours compared to PD-L1-negative tumours), but only with EFS, with 66% of patients with PD-L1-positive tumours being event-free at 1 year upon chemo-immunotherapy (versus 55% for PD-L1 negative) [14]. Nevertheless, both neoadjuvant-only and peri-operative strategies have reached impressive survival benefits, with a 5-year OS rate ranging from 65% to 80% for the SAKK 16/14 trial [16] and the Checkmate-159 trial [7], respectively, but clear interaction between pCR and OS did not emerge from randomized trials [15].

Nowadays, novel biomarkers to guide treatment allocation beyond PD-L1 expression are under investigation, such as circulating tumor DNA (ctDNA), which showed a promising activity in predicting disease recurrence [8], or multiomics algorithms, combining tumor-intrinsic, immune microenvironment, and systemic factors, supporting the development of precision immunotherapy strategies [17]. Unfortunately, these tools are still far from entering clinical practice and are therefore limited to research settings only.

### Challenges of the neoadjuvant phase: not only a surgical problem

#### Restaging and technical challenges

Overall, the recommended timing for surgery is between 4 and 6 weeks after the last cycle of neoadjuvant therapy [18]. However, adding immunotherapy to neoadjuvant PBC could raise some concerns in terms of adverse events, which might lead to postponed or even cancelled surgery. Of note, in randomized clinical trials, neoadjuvant chemo-immunotherapy does not seem to increase this risk if compared with PBC only. Namely, from 7% to 23% of patients across all trials dropped out of the surgical program, with the main reasons for cancellation being patient refusal (1–8.9%) and progressive disease (0–7.4%) [19].

Furthermore, radiologic restaging plays a crucial role in re-assessing the eligibility for surgery after neoadjuvant treatment. Restaging should be done with contrast-enhanced CT, while repeated invasive mediastinal staging is not recommended except when disease progression is suspected on imaging [18]. Nodal immune flare after immunotherapy might mimic disease progression and complicate the interpretation of CT-scans [20]; therefore, it is essential that the multidisciplinary team remains aware of this phenomenon and considers it when evaluating nodal enlargement after immunotherapy. Invasive techniques for mediastinal re-staging, such as EBUS or mediastinoscopy, should be considered when nodal progression is suspected, especially when progression leads to a non-resectable stage and significantly changes previously intended treatment.

From a technical point of view, neoadjuvant chemo-immunotherapy was assumed to complicate surgery and negatively influence surgical outcomes. Contrarily, randomized trials reported a higher rate of radical resections (R0), a higher rate of minimally invasive surgery and a lower rate of pneumonectomies for the immunotherapy arms [21–23]. Recent observational studies are trying to collect international experiences on real-world thoracic surgery after immunotherapy-based regimens, in order to standardize complexity definition. Due to hilar fibrosis and adhesions, surgical complexity could be increased and therefore the ‘surgical toolbox’ should offer techniques to deal with high complexity, such as reconstructive techniques and conversion to open surgery in case of minimally invasive surgical approaches [24]. Results from Nardini *et al.*, available as abstract at the European Lung Cancer Congress 2025, showed different factors that correlated with surgical complexity in this setting, such as cN2 disease, absence of nodal downstaging and PD-L1 expression ≥50% [25].

#### Borderline resectable and unresectable tumours in the immunotherapy era

Up to date, there are no trials with long-term results evaluating neoadjuvant chemo-immunotherapy for unresectable NSCLCs as a conversion strategy, and current guidelines state not to use neoadjuvant chemo-immunotherapy to attempt inducing resectability [18]. However, resectability is not clearly defined, and the European Organisation for the Treatment and Research of Cancer (EORTC) Lung Cancer Group (LCG) has launched an international Delphi process as an attempt to reach a standardized definition [26]. It is otherwise true that recent trials also include patients with multilevel N2 disease, which might be considered ‘upfront resectable’ or ‘borderline resectable’ according to surgical expertise, enhancing the possibility for these patients to receive a radical resection [22, 23]. For this particular subgroup, data on comparisons between the PACIFIC regimen (chemoradiotherapy followed by durvalumab [27]) or peri-operative chemo-immunotherapy plus surgery are missing, and the ongoing MDT-BRIDGE trial will investigate the role of sequential radiotherapy plus durvalumab consolidation in patients unable to proceed to surgery after neoadjuvant therapy [28].

A distinct subgroup among unresectable NSCLCs are the large volume and/or cavitating primary tumors, for whom complications (such as fatal hemorrhage or pulmonary abscesses) after chemoradiotherapy might occur more frequently [29]. Alternatives to standard of care chemoradiotherapy are being explored in this patient population, and the ongoing UPLAN trial is exploring the feasibility and safety of upfront resection of the primary tumor followed by chemoradiotherapy and durvalumab consolidation (NCT05620199).

#### Primary resistances upon neoadjuvant immunotherapy-based regimens

Despite the promising outcomes of neoadjuvant chemo-immunotherapy in resectable NSCLC, a subset of patients exhibits primary resistance: up to 7% of patients experience disease progression during neoadjuvant chemo-immunotherapy and become consequently ineligible for surgical resection [19]. Given the recent adoption of neoadjuvant immunotherapy-based regimens, evidence guiding management in such cases remains limited. If the disease remains suitable for local treatment (despite progression), definitive chemoradiotherapy followed by durvalumab—mirroring the standard for unresectable stage III NSCLC—may represent a rational approach [27]. However, data supporting this strategy post-neoadjuvant chemoimmunotherapy are lacking and it is not clear whether adjuvant durvalumab has any benefit in this situation. In a prospective registry, 86% of patients not undergoing surgery after neoadjuvant immunotherapy (36% of the total cohort) received radiotherapy, demonstrating the feasibility of alternative local treatments [30].

### New approaches: combinations and escalations

Multiple studies are investigating how to integrate new options in the neoadjuvant setting. In particular, the ongoing SAKK 16/18 non-comparative randomized phase II trial is evaluating low-dose, immunomodulatory radiotherapy combined with chemoimmunotherapy prior to resection, aiming to enhance immunotherapy efficacy [31]. Varying radiotherapy dose-fractionation schedules were applied, and their differential immunomodulatory effects will be analyzed in future exploratory work. Interim results indicated that surgery remained feasible without increased toxicity, however, final efficacy outcomes are pending. Furthermore, the non-randomized phase II SACTION-01 study applied stereotactic body radiotherapy to the primary tumor prior to chemoimmunotherapy to improve pathological response rates, with immature but interesting results (MPR rate of 80%) [32]. Similarly, in the SQUAT trial, patients with stage IIIA–B N2 NSCLC (TNM8) received concurrent chemoradiotherapy plus durvalumab followed by surgery and 1 year of adjuvant durvalumab: the MPR rate was 63% and the pCR rate was 23%, which could translate into improved survivals at a longer follow-up [33].

As far as new immunotherapeutic agents are concerned, the phase 2 NeoCOAST-2 study is evaluating multiple combinations in patients with resectable NSCLC, such as anti-CD73 monoclonal antibody (mAb), anti-NKG2A mAb, PD-1/CTLA-4 bispecific mAb and anti-TROP2 directed antibody drug-conjugate. Preliminary results show that treatments in all arms led to improvements in MPR rates along with a manageable safety profile and surgical rates, comparable to currently approved neoadjuvant and peri-operative immunotherapy-based regimens [34], but longer follow-up is needed.

## TYROSINE-KINASE INHIBITOR-BASED STRATEGIES

### Neoadjuvant-only strategy

The majority of neoadjuvant phase II trials focusing on EGFR inhibition via targeted therapies such as TKIs have shown encouraging results, but are still not routinely included in clinical practice. Neoadjuvant erlotinib in stage IIIA-N2 NSCLC resulted in an overall response rate (ORR) of 42% [35], and aneoadjuvant gefinitib in stage II–IIIA NSCLC achieved an ORR of 54.5%, but MPR was limited to 24.2% of patients [36]. Likewise, in the NEOS and NCT03433469 phase II trials, the third-generation EGFR-TKI osimertinib led to an ORR of 71% and 52%, but the MPR rate was only 10.7% and 16.7%, respectively [37, 38]. The ongoing phase III NeoADAURA trial is evaluating neoadjuvant osimertinib with or without chemotherapy in stage II–IIIB NSCLC, and it will probably provide essential information for the implementation of this strategy [39]. So far, these approaches did not translate in long-term survival benefits (mostly due to use of TKIs at first recurrence) and the MPR/pCR rates are still lower than with immunotherapy-based regimens.

### Adjuvant-only strategy

Nowadays, adjuvant TKIs are recommended by international guidelines. The phase III ADAURA trial demonstrated the efficacy of 3-year osimertinib in patients with resected stage IB-IIIA (TNM7) NSCLC harboring a common *EGFR* mutation (exon 19 deletion or L858R point-mutation). The trial reported a 4-year DFS of 73% versus 38%, and a 5-year OS rate of 88% versus 78% in the osimertinib and placebo arm, respectively. Overall, fewer local, regional, and distant recurrences were observed with osimertinib, which led to its recommendation as a standard-of-care for patients with *EGFR*-mutated stage IB-III resected NSCLC [40].

Following the success of ADAURA in *EGFR*-mutated tumours, the phase III ALINA trial evaluated 2 years of adjuvant alectinib versus standard PBC in patients with *ALK*-rearranged stage IB-IIIA (TNM7) resected NSCLC. Alectinib showed a significantly higher 3-year DFS (88.7%) compared to the standard arm (54%), and this benefit was confirmed across all subgroups. Despite OS not being reached yet, the American Food and Drug Administration and the European Medicines Agency have granted accelerated approval for alectinib for this specific population [41].

### Perioperative strategies

The perioperative setting has been only partially explored for patients with *EGFR*-mutant or *ALK*-rearranged tumours. The randomized phase 2 trial EMERGING-CTONG, evaluating 6 weeks of neoadjuvant plus 12 months of adjuvant erlotinib versus neoadjuvant PBC, showed no significant benefit for the TKI-arm in terms of ORR or pCR, and the benefit in PFS that was observed in the erlotinib group did not translate into an OS benefit [42]. The NORA trial (peri-operative osimertinib) was also negative for its primary end-point (ORR) [43].

On the other hand, for *ALK*-rearranged tumours, the interim analysis of the ALNEO-GOIRC-01-2020 phase II trial in resectable stage III *ALK*-positive NSCLC reported that 8 weeks of neoadjuvant alectinib plus 96 weeks of adjuvant treatment resulted in an ORR of 68%, an MPR rate of 38.9%, and a pCR rate of 17%, supporting future developments in this setting [44].

Many questions are still to be answered for patients with early-stage oncogene-addicted tumours. Firstly, the evidence is currently limited for any other oncogenic driver except for *EGFR*-mutations and *ALK*-rearrangements. Moreover, combination strategies (i.e. with PBC) are under evaluation to explore their role in improving MPR/pCR rates, and possibly translate into longer survival benefits. Finally, the optimal duration of the adjuvant phase with TKIs is unknown, differing greatly across trials. Real-time monitoring of ctDNA is a promising tool for tailoring adjuvant treatment, given the established role of molecular residual disease in predicting DFS after radical surgery for *EGFR*-mutated tumours [45], but it is still far from being implemented in clinical practice, as discussed before.

## CONCLUSION AND FUTURE DIRECTIONS

Many new treatment options are now available for early-stage NSCLC, but biomarkers that can guide clinicians towards tailored choices, beyond *EGFR* and *ALK*, are still lacking. Nevertheless, it has clearly emerged that multidisciplinary management of patients with early-stage resectable NSCLC in routine clinical practice is crucial to define the optimal, evidence-based and patient-centric treatment strategy. Shared decisions should take into account many different factors, both patient-based and tumor-based, such as patients’ preferences, expected tolerability of different regimens, PD-L1 expression and lymph node involvement. Ongoing and future trials will continue to refine personalized treatment approaches to improve survival and quality of life.

## References

[1] Postmus PE , Kerr KM, Oudkerk M et al; ESMO Guidelines Committee. Early and locally advanced non-small-cell lung cancer (NSCLC): ESMO Clinical Practice Guidelines for diagnosis, treatment and follow-up. Ann Oncol 2017;28:iv1–iv21. 10.1093/annonc/mdx222 28881918

[2] Chang JY , Mehran RJ, Feng L et al; STARS Lung Cancer Trials Group. Stereotactic ablative radiotherapy for operable stage I non-small-cell lung cancer (revised STARS): long-term results of a single-arm, prospective trial with prespecified comparison to surgery. Lancet Oncol 2021;22:1448–57. 10.1016/S1470-2045(21)00401-0 34529930 PMC8521627

[3] de Ruiter JC , van Diessen JNA, Smit EF, ESLUNG group et al ‘Minimally invasive lobectomy versus stereotactic ablative radiotherapy for stage I non-small cell lung cancer’. Eur J Cardio-Thorac Surg 2022;62. 10.1093/ejcts/ezac118 35348664

[4] Saji H , Okada M, Tsuboi M et al; West Japan Oncology Group and Japan Clinical Oncology Group. Segmentectomy versus lobectomy in small-sized peripheral non-small-cell lung cancer (JCOG0802/WJOG4607L): a multicentre, open-label, phase 3, randomised, controlled, non-inferiority trial. Lancet 2022;399:1607–17. 10.1016/S0140-6736(21)02333-3 35461558

[5] Altorki N , Wang X, Damman B et al Lobectomy, segmentectomy, or wedge resection for peripheral clinical T1aN0 non–small cell lung cancer: a post hoc analysis of CALGB 140503 (Alliance). J Thorac Cardiovasc Surg 2024;167:338–47.e1. 10.1016/j.jtcvs.2023.07.008 37473998 PMC10794519

[6] Forde PM , Chaft JE, Smith KN et al Neoadjuvant PD-1 blockade in resectable lung cancer. N Engl J Med 2018;378:1976–86. 10.1056/NEJMoa1716078 29658848 PMC6223617

[7] Rosner S , Reuss JE, Zahurak M et al Neoadjuvant nivolumab in early-stage non–small cell lung cancer (NSCLC): five-year outcomes. JCO 2022;40:8537. 10.1200/JCO.2022.40.16_suppl.8537

[8] Forde PM , Spicer J, Lu S et al; May CheckMate 816 Investigators. Neoadjuvant nivolumab plus chemotherapy in resectable lung cancer. N Engl J Med 2022;386:1973–85. 10.1056/NEJMoa2202170 35403841 PMC9844511

[9] Forde P, Spicer J, Provencio M et al. Overall Survival with Neoadjuvant Nivolumab plus Chemotherapy in Lung Cancer. N Eng J Med 2025 Jun 2. 10.1056/NEJMoa2502931. Online ahead of print. 40454642

[10] Felip E , Altorki N, Zhou C et al; IMpower010 Investigators. Adjuvant atezolizumab after adjuvant chemotherapy in resected stage IB–IIIA non-small-cell lung cancer (IMpower010): a randomised, multicentre, open-label, phase 3 trial. Lancet 2021;398:1344–57. 10.1016/S0140-6736(21)02098-5 34555333

[11] Wakelee HA , Altorki NK, Zhou C et al IMpower010: final disease-free survival (DFS) and second overall survival (OS) interim results after ≥5 years of follow up of a phase III study of adjuvant atezolizumab vs best supportive care in resected stage IB-IIIA non-small cell lung cancer (NSCLC). JCO 2024;42:LBA8035–LBA8035. 10.1200/JCO-24-01681

[12] O’Brien M , Paz-Ares L, Marreaud S et al Pembrolizumab versus placebo as adjuvant therapy for completely resected stage IB–IIIA non-small-cell lung cancer (PEARLS/KEYNOTE-091): an interim analysis of a randomised, triple-blind, phase 3 trial. Lancet Oncol 2022;23:1274–86. 10.1016/S1470-2045(22)00518-6 36108662

[13] Goss G , Darling GE, Westeel V et al LBA48 CCTG BR.31: a global, double-blind placebo-controlled, randomized phase III study of adjuvant durvalumab in completely resected non-small cell lung cancer (NSCLC). Ann Oncol 2024;35:S1238. 10.1016/j.annonc.2024.08.2289

[14] Nuccio A , Viscardi G, Salomone F et al Systematic review and meta-analysis of immune checkpoint inhibitors as single agent or in combination with chemotherapy in early-stage non-small cell lung cancer: impact of clinicopathological factors and indirect comparison between treatment strategies. Eur J Cancer 2023;195:113404. 10.1016/j.ejca.2023.113404 37948842 PMC12697757

[15] Hines JB , Cameron RB, Esposito A et al Evaluation of major pathologic response and pathologic complete response as surrogate end points for survival in randomized controlled trials of neoadjuvant immune checkpoint blockade in resectable in NSCLC. J Thorac Oncol 2024;19:1108–16. 10.1016/j.jtho.2024.03.010 38461929 PMC12697759

[16] Rothschild S, Zippelius A, Hayoz S et al. Perioperative durvalumab in addition to neoadjuvant chemotherapy in patients with stage IIIa(N2) non-small cell lung cancer: Final analysis of the trial SAKK 16/14. J Thorac Oncol 2025;20:S123-S150. 10.1016/S1556-0864(25)00632-X

[17] Vaccaro A , Rahal Z, Kadara H, Cascone T. A roadmap to precision immunotherapy for early-stage non–small cell lung cancer. Cancer Discov 2025;15:884–9. 10.1158/2159-8290.CD-25-0262 39997992 PMC13273914

[18] Spicer JD , Cascone T, Wynes MW et al Neoadjuvant and adjuvant treatments for early stage resectable NSCLC: consensus recommendations from the International Association for the Study of Lung Cancer. J Thorac Oncol 2024;19:1373–414. 10.1016/j.jtho.2024.06.010 38901648

[19] Sorin M , Prosty C, Ghaleb L et al Neoadjuvant chemoimmunotherapy for NSCLC. JAMA Oncol 2024;10:621–33. 10.1001/jamaoncol.2024.0057 38512301 PMC10958389

[20] Cascone T , Weissferdt A, Godoy MCB et al Nodal immune flare mimics nodal disease progression following neoadjuvant immune checkpoint inhibitors in non-small cell lung cancer. Nat Commun 2021;12:5045. 10.1038/s41467-021-25188-0 34413300 PMC8376947

[21] Heymach JV , Harpole D, Mitsudomi T et al; AEGEAN Investigators. Perioperative durvalumab for resectable non–small-cell lung cancer. N Engl J Med 2023;389:1672–84. 10.1056/NEJMoa2304875 37870974

[22] Cascone T , Awad MM, Spicer JD et al; May CheckMate 77T Investigators. Perioperative nivolumab in resectable lung cancer. N Engl J Med 2024;390:1756–69. 10.1056/NEJMoa2311926 38749033

[23] Wakelee H , Liberman M, Kato T et al; KEYNOTE-671 Investigators. Perioperative pembrolizumab for early-stage non–small-cell lung cancer. N Engl J Med 2023;389:491–503. 10.1056/NEJMoa2302983 37272513 PMC11074923

[24] Trabalza Marinucci B , Mancini M, Siciliani A et al Surgical techniques for non-small-cell lung cancer after neoadjuvant chemo-immunotherapy: state of art and review of the literature. Cancers (Basel) 2025;17:638. 10.3390/cancers17040638 40002233 PMC11853686

[25] Nardini M, Lodhia J, Tcherveniakov P et al. A multifactorial score to predict surgical complexity of lung resection following neoadjuvant chemo-immunotherapy. J Thorac Oncol 2025;20:S123–S150. 10.1016/S1556-0864(25)00632-X

[26] Dingemans A-M , Remon J, Hendriks L et al OA06.05 consensual definition of stage III NSCLC resectability: EORTC-Lung Cancer Group Initiative with Other Scientific Societies. J Thorac Oncol 2023;18:S57–S58. 10.1016/j.jtho.2023.09.046

[27] Antonia SJ , Villegas A, Daniel D et al; PACIFIC Investigators. Durvalumab after chemoradiotherapy in stage III non–small-cell lung cancer. N Engl J Med 2017;377:1919–29. 10.1056/NEJMoa1709937 28885881

[28] Reck M , Nadal E, Girard N et al MDT-BRIDGE: neoadjuvant durvalumab plus chemotherapy followed by either surgery and adjuvant durvalumab or chemoradiotherapy and consolidation durvalumab in resectable or borderline-resectable stage IIB–IIIB NSCLC. Clin Lung Cancer 2024;25:587–93.e3. 10.1016/j.cllc.2024.06.007 39003185

[29] Phernambucq ECJ , Hartemink KJ, Smit EF et al Tumor cavitation in patients with stage III non–small-cell lung cancer undergoing concurrent chemoradiotherapy: incidence and outcomes. J Thorac Oncol 2012;7:1271–5. 10.1097/JTO.0b013e3182582912 22659960

[30] Ay L , Steiner D, Fabikan H et al Neoadjuvant therapy in early-stage non-small cell lung cancer: a real-world analysis. Lung Cancer 2024;198:107997. 10.1016/j.lungcan.2024.107997 39486111

[31] Mauti LA , Finazzi T, Holer L et al SAKK 16/18: immune-modulatory radiotherapy to enhance the effects of neoadjuvant PD-L1 blockade after neoadjuvant chemotherapy in patients with resectable stage III (N2) non-small cell lung cancer (NSCLC)—A multicenter phase II trial. JCO 2023;41:8547. 10.1200/JCO.2023.41.16_suppl.8547

[32] Zhao Z-R , Liu S-L, Zhou T et al Stereotactic body radiation therapy with sequential immunochemotherapy as neoadjuvant therapy in resectable non-small cell lung cancer (SACTION-01 study). JCO 2023;41:8540. 10.1200/JCO.2023.41.16_suppl.8540

[33] Hamada A et al Neoadjuvant concurrent chemo-immuno-radiation therapy followed by surgery and adjuvant immunotherapy for resectable stage III N2 non-small cell lung cancer: primary results from SQUAT trial (WJOG 12119L). J Thorac Oncol 2025. Apr 9:S1556-0864(25)00656-2. 10.1016/j.jtho.2025.03.051. Online ahead of print. 40216327

[34] Cascone T , Florian G, Bonanno L et al PL02.07 neocoast-2: efficacy and safety of neoadjuvant durvalumab (D) + novel anticancer agents + CT and adjuvant D ± novel agents in resectable NSCLC. J Thorac Oncol 2024;19:S1–S2. 10.1016/j.jtho.2024.09.013

[35] Xiong L , Li R, Sun J et al Erlotinib as neoadjuvant therapy in stage IIIA (N2) *EGFR* mutation-positive non-small cell lung cancer: a prospective, single-arm, phase II study. Oncologist 2019;24:157–e64. no. Feb. 10.1634/theoncologist.2018-0120 30158288 PMC6369937

[36] Zhang Y , Fu F, Hu H et al Gefitinib as neoadjuvant therapy for resectable stage II-IIIA non–small cell lung cancer: a phase II study. J Thorac Cardiovasc Surg 2021;161:434–42.e2. 10.1016/j.jtcvs.2020.02.131 32340810

[37] Lv C , Fang W, Wu N et al Osimertinib as neoadjuvant therapy in patients with EGFR-mutant resectable stage II-IIIB lung adenocarcinoma (NEOS): a multicenter, single-arm, open-label phase 2b trial. Lung Cancer 2023;178:151–6. 10.1016/j.lungcan.2023.02.011 36863124

[38] Blakely CM , Urisman A, Gubens MA et al Neoadjuvant osimertinib for the treatment of stage I-IIIA epidermal growth factor receptor–mutated non–small cell lung cancer: a phase II multicenter study. J Clin Oncol 2024;42:3105–14. 10.1200/JCO.24.00071 39028931 PMC11379363

[39] Tsuboi M , Weder W, Escriu C et al Neoadjuvant osimertinib with/without chemotherapy versus chemotherapy alone for *EGFR*-mutated resectable non-small-cell lung cancer: neoADAURA. Future Oncol 2021;17:4045–55. 10.2217/fon-2021-0549 34278827 PMC8530153

[40] Tsuboi M , Herbst RS, John T et al Overall survival with osimertinib in resected *EGFR* -mutated NSCLC. N Engl J Med 2023;389:137–47. 10.1056/NEJMoa2304594 37272535

[41] Wu Y-L , Dziadziuszko R, Ahn JS et al Alectinib in resected *ALK*-positive non–small-cell lung cancer. N Engl J Med 2024;390:1265–76. 10.1056/NEJMoa2310532 38598794

[42] Zhong W-Z , Yan H-H, Chen K-N et al Erlotinib versus gemcitabine plus cisplatin as neoadjuvant treatment of stage IIIA-N2 EGFR-mutant non-small-cell lung cancer: final overall survival analysis of the EMERGING-CTONG 1103 randomised phase II trial. Signal Transduct Target Ther 2023;8:76. 10.1038/s41392-022-01286-3 36823150 PMC9950485

[43] Lee JB , Choi S-J, Shim HS et al Neoadjuvant and adjuvant osimertinib in stage IA to IIIA, EGFR-mutant NSCLC (NORA). J Thorac Oncol 2025;20:641–50. 10.1016/j.jtho.2024.12.023 39732365

[44] Leonetti A , Boni L, Gnetti L et al MA01.03 neoadjuvant alectinib in potentially resectable stage III ALK-positive NSCLC: interim analysis of ALNEO-GOIRC-01-2020 phase II trial. J Thorac Oncol 2024;19:S52. 10.1016/j.jtho.2024.09.091

[45] Herbst RS , John T, Grohé C et al Molecular residual disease analysis of adjuvant osimertinib in resected EGFR-mutated stage IB–IIIA non-small-cell lung cancer. Nat Med 2025;31:1958–68. 10.1038/s41591-025-03577-y 40097663 PMC12176615

